# All-Trans Retinoic Acid Enhances both the Signaling for Priming and the Glycolysis for Activation of NLRP3 Inflammasome in Human Macrophage

**DOI:** 10.3390/cells9071591

**Published:** 2020-07-01

**Authors:** Ahmad Alatshan, Gergő E. Kovács, Azzam Aladdin, Zsolt Czimmerer, Krisztina Tar, Szilvia Benkő

**Affiliations:** 1 Departments of Physiology, Faculty of Medicine, University of Debrecen, H-4012 Debrecen, Hungary; ahmad.alatshan@med.unideb.hu (A.A.); kovacs.gergo@med.unideb.hu (G.E.K.); 2 Doctoral School of Molecular Cellular and Immune Biology, Faculty of Medicine, University of Debrecen, H-4012 Debrecen, Hungary; 3 Department of Medical Chemistry, Faculty of Medicine, University of Debrecen, H-4032 Debrecen, Hungary; aladdin@med.unideb.hu (A.A.); tark@med.unideb.hu (K.T.); 4 Doctoral School of Molecular Medicine, University of Debrecen, H-4032 Debrecen, Hungary; 5 Department of Biochemistry and Molecular Biology, Faculty of Medicine, University of Debrecen, H-4032 Debrecen, Hungary; czimmerer.zsolt@med.unideb.hu

**Keywords:** all-trans retinoic acid, NLRP3 inflammasome, IL-1β, signaling, metabolism, human macrophages

## Abstract

All-trans retinoic acid (ATRA) is a derivative of vitamin A that has many important biological functions, including the modulation of immune responses. ATRA actions are mediated through the retinoic acid receptor that functions as a nuclear receptor, either regulating gene transcription in the nucleus or modulating signal transduction in the cytoplasm. NLRP3 inflammasome is a multiprotein complex that is activated by a huge variety of stimuli, including pathogen- or danger-related molecules. Activation of the inflammasome is required for the production of IL-1β, which drives the inflammatory responses of infectious or non-infectious sterile inflammation. Here, we showed that ATRA prolongs the expression of IL-6 and IL-1β following a 2-, 6-, 12-, and 24-h LPS (100ng/mL) activation in human monocyte-derived macrophages. We describe for the first time that ATRA modulates both priming and activation signals required for NLRP3 inflammasome function. ATRA alone induces NLRP3 expression, and enhances LPS-induced expression of NLRP3 and pro-IL-1β via the regulation of signal transduction pathways, like NF-κB, p38, and ERK. We show that ATRA alleviates the negative feedback loop effect of IL-10 anti-inflammatory cytokine on NLRP3 inflammasome function by inhibiting the Akt-mTOR-STAT3 signaling axis. We also provide evidence that ATRA enhances hexokinase 2 expression, and shifts the metabolism of LPS-activated macrophages toward glycolysis, leading to the activation of NLRP3 inflammasome.

## 1. Introduction

IL-1β is a master cytokine that plays an important role in many immunological and physiological processes [1]. As a conductor cytokine, it regulates the activation of cells and modulates cytokine production. Additionally, it has an important role in T helper (Th) cell polarization, connecting innate and adaptive immune responses. The production of IL-1β is tightly regulated by multiprotein complexes called inflammasomes. The NOD-, LRR- and pyrin domain-containing protein 3 (NLRP3) inflammasome is a well-characterized inflammasome, and unlike other inflammasome complexes, it can be activated by a wide range of stimuli. Importantly, NLRP3 inflammasome-mediated IL-1β is required to drive an effective inflammatory response in infectious diseases triggered by pathogenic microorganisms. Additionally, NLRP3 inflammasome-derived IL-1β is also the major driver of chronic low-grade sterile inflammation that accompanies conditions, such as metabolic syndromes and aging, endometriosis, and bronchiolitis obliterans syndrome, eventually leading to tissue damage [2,3,4,5]. For this reason, the pharmacological manipulation of NLRP3 inflammasome has become the focus of intensive studies and medical trials [6,7,8].

For safety reasons, in order to avoid unwanted activation, NLRP3-mediated IL-1β secretion requires two signals. The first priming signal is initiated by extracellular stimuli mediated by Toll-like receptors (TLRs) or cytokine receptors [9]. These stimuli activate a variety of signal transduction pathways, including NF-κB, p38, and ERK, which are required for the transcription of the inflammasome response-related genes, mainly NLRP3 and IL-1β. Furthermore, the priming signal is also needed for the expression and licensing of NLRP3 through post-translational modification [10,11]. A second signal, which may derive from a variety of PAMPs or DAMPs, is required for NLRP3 inflammasome oligomerization as part of activation. Subsequently, licensed NLRP3 protein interacts with ASC, which recruits pro-caspase-1, and the activation of the enzyme leads to the maturation of IL-1β cytokine [12].

Nuclear receptors (NRs) are transcription factors that are activated by lipid-soluble endogenous or exogenous ligands, and, among many functions, they are important modulators of inflammatory responses [13,14,15]. One of their family members is the retinoic acid receptor (RAR), which forms heterodimer with retinoic X receptor (RXR), and mediates the effect of retinoic acid [14,16]. Retinoic acid (RA) is an active form of vitamin A derivative, and can be generated in multiple forms; however, all-trans RA (ATRA) is the most abundant and active isomer in the human body [17]. Upon activation, cytosolic RAR translocates to the nucleus and binds to conserved RA-responsive elements (RAREs), regulating the transcription of several target genes [17,18]. Besides the nuclear function, ligand-bound RAR also has non-genomic activity in the cytosol, as it binds and regulates the proteins of signal transduction cascades, such as Akt, ERK, or p38 [19,20,21]. 

ATRA is known to play a crucial role in many biological processes, such as cell proliferation, differentiation, tumorigenesis, as well as inflammation [22,23]. While the role of ATRA in maintaining the proper mucosal homeostasis and immunological tolerance of mucosal immune cells has been extensively studied [17,24], less is known about its effect on non-mucosal immune cells, such as monocyte-derived macrophages (MΦs). It has been shown that vitamin A deficiency is characterized by increased susceptibility to infectious diseases, and supplementation with vitamin A and related retinoids improved immune responses in many ways, including the activation of myeloid cells and changes in cytokine production [17,25,26,27]. Though the nature and detailed mechanisms of the ATRA effect are still not clear—and due to the various model systems, it may be controversial—studies have provided solid evidence on the modulatory role of ATRA on inflammatory cytokine production. Importantly, it was reported that ATRA enhanced IL-1β secretion in cells, such as mast cells of the skin [28]; alveolar macrophages infected by *Mycobacterium tuberculosis* [29]; human PBMCs [30]; an LPS-induced THP-1 monocytic cell line [31]; or in PMA-stimulated human monocytes [32]. However, while NLRP3 inflammasome has a central regulatory role in IL-1β production in myeloid cells, and the importance of NLRP3 inflammasome-mediated inflammatory responses has been shown in many infectious diseases [33], the possible effects of ATRA on the NLRP3 inflammasome-mediated IL-1β production and the related regulatory mechanisms are yet to be characterized. 

In our study, we aimed to investigate the potential modulatory effect of ATRA on monocyte-derived MΦs, a well-characterized macrophage subtype that is a commonly used model for NLRP3 inflammasome-mediated IL-1β production. Here, we show for the first time that ATRA treatment significantly modulates both the priming and the activation of NLRP3 inflammasome of LPS-activated cells. We show enhanced expression of NLPR3 and pro-IL-1β expression. Additionally, we show that ATRA shifts the metabolism toward glycolysis, and provide evidence for the potential mechanisms that, in part, augments NLRP3 inflammasome activity.

## 2. Materials and Methods

### 2.1. Reagents

MCC950 (NLRP3-selective inflammasome inhibitor) and Ultrapure LPS from *Escherichia coli* were obtained from InvivoGen (San Diego, CA, USA). All-trans retinoic acid, adenosine triphosphate (ATP), and 3-bromopyruvate (3BP) were purchased from Sigma-Aldrich (St. Louis, MO, USA). Recombinant human IL-10 was obtained from PeproTech (Rocky Hill, NJ, USA).

### 2.2. Ethics Statement, and Monocyte Isolation and Differentiation

Leukocyte-enriched buffy coats were obtained from healthy donors. The donors provided written informed consent. The procedure was documentary approved by the Director of the National Blood Transfusion Service. The study and all experimental protocols were in accordance with, and documentary approved by the Regional and Institutional Ethics Committee of the University of Debrecen (Debrecen, Hungary). 

### 2.3. Monocyte Isolation and Macrophage Differentiation

Human peripheral blood mononuclear cells (PBMCs) were isolated from leukocyte-enriched buffy coats. Briefly, the blood samples were diluted two-fold in physiological saline solution (PSS). The diluted blood was submitted to density-gradient centrifugation (1500 rpm, 10 min, at 18 ℃) using Ficoll Paque PLUS (GE Healthcare Life Sciences, Little Chalfont, United Kingdom). The PBMC layer was collected and washed twice with PSS and one time with MACS buffer (phosphate-buffered saline (PBS), 0.5% bovine serum albumin (BSA), and 2mM EDTA). Monocytes were purified from PBMCs using immunomagnetic positive selection with anti-CD14-conjugated microbeads according to the manufacturer’s instruction (Miltenyi Biotec, Bergisch Gladbach, Germany). After isolation, the cell density was determined and the freshly isolated monocytes were suspended in RPMI 1640 medium (Sigma-Aldrich, St. Louis, MO, USA) supplemented with 2 mM L-glutamine, 10% heat-inactivated FCS, and 500 U/mL of penicillin-streptomycin (Thermo Fisher Scientific, Waltham, MA, USA). Finally, the suspended monocytes were cultured in 24-well plates at a density of 1.5 × 106 cells/mL in 50 ng/mL M-CSF (PeproTech, Rocky Hill, NJ, USA) containing media and incubated at 37 °C and 5% CO_2_. After 48 h, half of the culture media was carefully removed and replaced with fresh media containing the same amounts of M-CSF. On day 5, the cells were used for the experiments.

### 2.4. Macrophage Treatment

After 5 days, the macrophages were treated with ATRA (1 µM) alone or pretreated with ATRA for 4 h and stimulated with LPS (100 ng/mL) for different time points. For IL-1β induction, macrophages were treated with ATP (5 mM) for 45 min. Where indicated, cultures were pretreated with an inhibitor for 1 h, and then LPS was applied. The control (mock) was treated with 0.1% DMSO/ethanol.

### 2.5. RNA Preparation, RT-PCR, and Quantitative Real-Time PCR

The otal RNA content was extracted using TriReagent (Molecular Research Center, Inc., Cincinnati, OH, USA) according to the manufacturer’s instructions. The RNA concentration and quality were determined by a spectrophotometer (NanoDrop ND1000; Promega Biosciences, Madison, WI, USA). The isolated RNA was treated with DNase and RNase inhibitor (Ambion, Austin, TX, USA). cDNA synthesis was done using random hexamers and the SuperScript II First-strand Reverse Transcriptase system (Thermo Fisher Scientific, Waltham, MA, USA).

### 2.6. Quantitative Real-Time PCR

For quantitative RT-PCR, Taqman Gene Expression Assays were used with the Taqman™ Gene Expression Master Mix (Applied Biosystems, Foster City, CA, USA). The amplification was performed using a QuantStudio12K Flex qPCR instrument (ABI). Human Taqman gene expression assays were purchased from Thermo Fisher Scientific (Waltham, MA, USA), NLRP3 (Hs00918082_m1), and IL-1β (Hs01555410_m1). The amplification program was, 10 min at 95 °C followed by 40 cycles of 10 s at 95 °C, and 1 min at 60 °C. The relative expression values for each transcript of interest were calculated by the comparative Ct method, and human cyclophilin (Ppia) was used for normalization.

### 2.7. Western Blot Analysis

After harvesting, the cells were washed with PBS; directly lysed in 2X Laemmli sample buffer (62.5 mM Tris-HCl (pH 6.8), containing 25% glycerol, 2% SDS, 1% b-mercaptoethanol, and 1% bromophenol blue); and boiled for 10 min. Proteins were separated by SDS-PAGE and transferred onto nitrocellulose membrane (Thermo Fisher Scientific, Waltham, MA, USA). The membrane was blocked with 5% non-fat dry milk diluted in TBS-Tween buffer (50 mM Tris, 0.5 M NaCl and 0.05% Tween-20, pH 7.6). The membrane was incubated overnight at 4 °C with primary antibodies in 1:1000 dilution. ASC (sc-30153) Abs were from Santa Cruz Biotechnology (Santa Cruz, CA, USA), NLRP3 (Cat. No. 15101), caspase-1 (Cat. No. 3866), IL-1β (Cat. No. 12703), cleaved caspase-1 (Cat. No. 4199), cleaved-IL-1β (Cat. No. 83186), p-Akt/(S473) (Cat. No. 9271), p-mTOR (Ser2448) (Cat. No. 2971), p-p70S6 Kinase (Thr389) (Cat. No. 9234), p70S6 Kinase (Cat. No. 2708), p-Stat3 (Tyr705) (Cat. No. 9145), p-p38 MAPK (Thr180/Tyr182) (Cat. No. 9211), p-SAPK/JNK (Thr183/Tyr185) (Cat. No. 9251), p-p44/42 MAPK (Erk1/2) (Thr202/Tyr204) (Cat. No. 9101), and p-IκBα (Ser32) (Cat. No. 2859) were obtained from Cell Signaling technology (Danvers, MA, USA). After the washing step, the membrane was incubated for 1 h at room temperature with a corresponding HRP-conjugated secondary Abs in 1:5000 dilution (goat anti-rabbit IgG, No. 170-6515) from Bio-Rad Laboratories (Hercules, CA, USA). Membrane-bound peroxidase proteins were detected on X-ray films using the ECL system (SuperSignal West Pico/Femto chemiluminescent substrate; (Thermo Fisher Scientific, Waltham, MA, USA). β-Actin (8457) (Cell Signaling technology, Danvers, MA, USA) was used as the internal control.

### 2.8. Metabolic Assays and Extracellular Flux Analysis

Real-time changes in the extracellular acidification rate (ECAR) and oxygen consumption rate (OCR) of macrophages were performed using a Seahorse XF 96 Analyzer (Seahorse Biosciences, North Billerica, MA, USA). Briefly, isolated monocytes (50,000 cell/well) were plated and differentiated in Seahorse XF96 cell culture microplates (Seahorse Biosciences, North Billerica, MA, USA). Then, macrophages were treated as described above and subjected to the metabolic assays. For the mitochondrial stress test, cells were washed and incubated in XF assay medium (Seahorse Bioscience, North Billerica, MA, USA) supplemented with 10 mM glucose and 2 mM L-glutamine and incubated for one hour at 37 °C in a CO_2_-free incubator. The bassline OCR was recorded, and the cells were then subjected to the following compounds: Oligomycin (Oligo), an ATP synthetase inhibitor (1 μM); carbonyl cyanide-4-(trifluoromethoxy) phenylhydrazone (FCCP), an uncoupling agent (1 μM); and rotenone and antimycin A (R + A) as mitochondrial complex I and III inhibitors (1:1 μM), respectively. Real-time changes in the OCR were recorded every 6 min (1 min mixing, 5 min measurement) for five loops. 

For the glycolytic stress test, the RPMI media was replaced by XF media supplemented with 2 mM L-glutamine and incubated for 1 h at 37 °C in CO_2_-free conditions. After equilibration, the real-time changes in the ECAR were recorded every 9 min (1 min mixing, 8 min measure) for 5 loops, during sequential treatment of the following compounds: 10 mM glucose (Glu), 1 µM oligomycin (Oligo), and 50 mM 2-deoxy-D-glucose (2-DG). The background control was determined by the testing media. The test was run for 90 min following the manufacturer’s protocol and the injection time for each compound is indicated in the graphs. The protein concentration was determined using the Bradford protein assay. The obtained values were normalized to the corresponding total protein content. Wave 2.3 Agilent Seahorse Desktop software was used for the data analysis.

### 2.9. Cytokine Measurements

To determine the concentration of IL-1β, IL-6, IL-10, and TNF-α in the cell culture supernatants, commercial enzyme-linked immunosorbent assay (ELISA) kits (BD Biosciences, San Diego, CA, USA) were used according to the manufacturer’s instructions. The minimum detection limits of the kits were 0.8 pg/mL for IL-1β, 2.2 pg/mL for IL-6, and 2pg/mL for IL-10 and TNF-α. Quantifications were performed by a FlexStation 3 Microplate Reader (Molecular Devices, Sunnyvale, CA, USA).

### 2.10. Statistical Analysis 

Experimental results are presented as the mean ± standard deviation (SD). Statistical significance was determined by analysis of variance (ANOVA) followed by the Tukey–Kramer test. Differences between groups were considered significant at *p* values of <0.05.

## 3. Results

### 3.1. ATRA Modifies LPS-Induced Proinflammatory Cytokine Secretion in Human Macrophages

**Figure 1:**
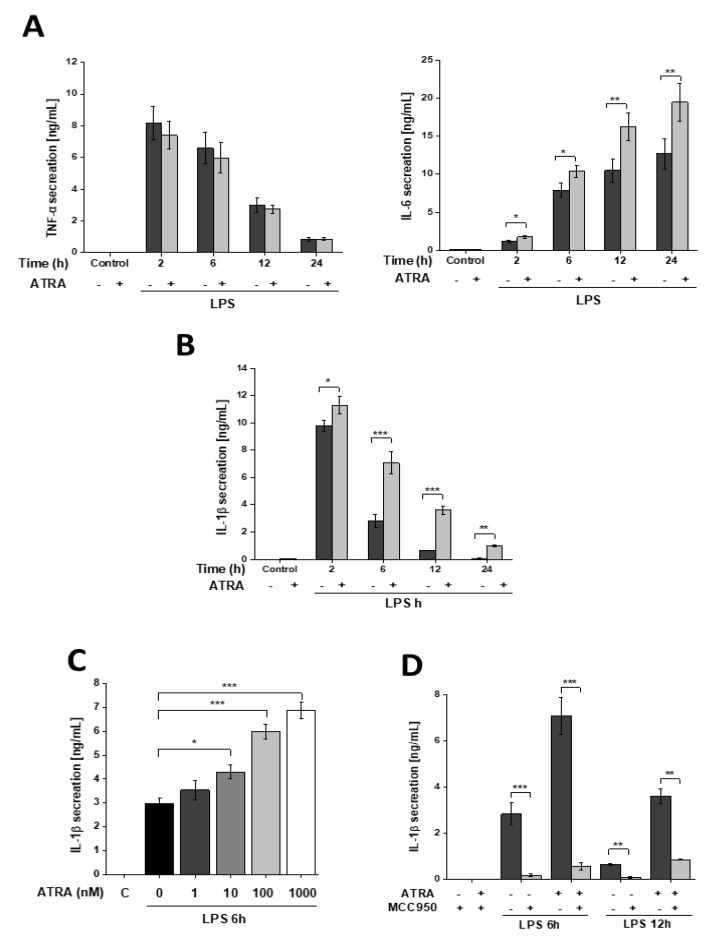
ATRA differentially modulates proinflammatory cytokine secretion of LPS-activated MΦs. MΦs were pre-incubated with ATRA (1 µM) where indicated, and then stimulated with LPS (100 ng/mL) for the indicated time. Cell culture supernatants were collected, and the secretion of (**A**) TNFα and IL-6 were measured by ELISA. (**B**) For IL-1β induction, cells were subsequently incubated with ATP (5 mM) for 45 min. (**C**) Cells were pre-treated with increasing concentrations of ATRA as indicated, and IL-β secretion was measured 6 h following treatment. (**D**) Cells were pretreated with MCC950 (1 µM) 1 h before ATP treatment. C, control (6 h mock-treated cells). Data were obtained from at least four healthy donors. All results are shown as means ± SEM. (* *p* < 0.05, ** *p* < 0.01, *** *p* < 0.001). +, −, presence or absence of indicated substance, respectively

To determine whether ATRA affects proinflammatory cytokine secretion of activated human monocyte-derived macrophages (MΦs), cells were treated with LPS in the absence or presence of ATRA for various time intervals, and cytokine production was measured using the ELISA method. We found that ATRA treatment had no effect on the TNFα secretion of resting and LPS-activated MΦs (Figure 1A); however, it significantly elevated the IL-6 secretion of LPS-treated cells. 

As we reported previously, LPS treatment results in rapid IL-1β secretion that reaches a peak at 2 h and then gradually decreases over time [34]. ATRA alone did not have an effect on IL-1β secretion; however, LPS-induced IL-1β secretion was significantly enhanced and prolonged by ATRA (Figure 1B). Furthermore, IL-1β secretion showed a positive correlation with the concentration of applied ATRA (Figure 1C). Treatment of the cells with the NLRP3 inhibitor MCC950 abolished IL-1β secretion (Figure 1D), indicating that the effect of ATRA on IL-1β production is mediated through an NLRP3 inflammasome-dependent pathway.

### 3.2. ATRA Prolongs LPS-Induced IL-1β Cytokine Secretion in Part by Augmenting LPS-Induced NLRP3 and Pro-IL-1β Expression

**Figure 2:**
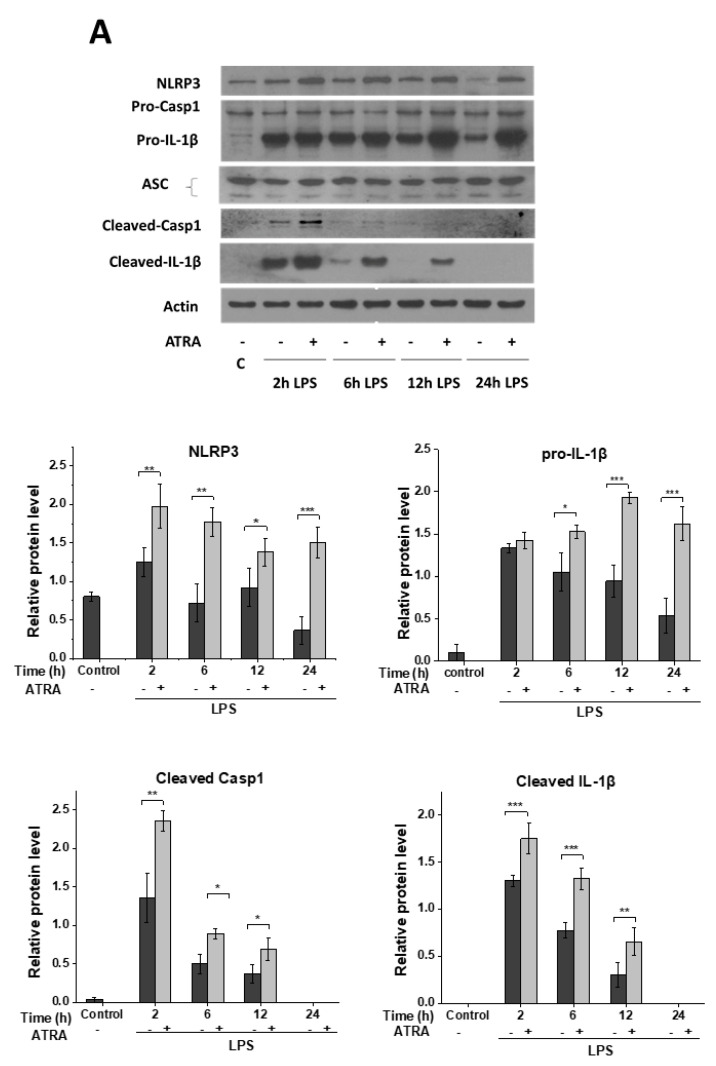

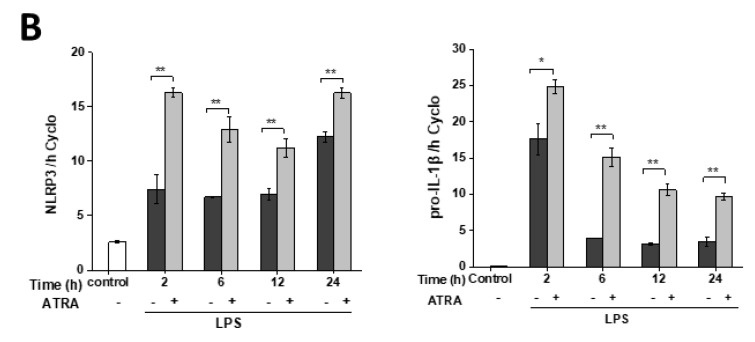
ATRA enhances LPS-induced pro-IL-1β and NLRP3 expression in human MΦs. MΦs were pre-incubated with ATRA (1 µM) where indicated prior to stimulation with LPS (100 ng/mL), then 5 mM ATP was applied for 45 min. (**A**) Representative immunoblot of NLRP3, pro-caspase-1, pro-IL-1β, and ASC from the cell lysates; and released caspase-1 and IL-1β in supernatant. Bar graphs represent the relative protein expression of pro-IL-1β and NLRP3 determined by densitometry. β-actin was used as the internal control. (**B**) The relative gene expression of pro-IL-1β and NLRP3 was measured by qPCR. The expression was normalized to the reference gene (human cyclophilin; Cyclo) expression. Data were obtained from at least four healthy donors. C, control (6 h mock-treated cells). All results are shown as means ± SEM. (* *p* < 0.05, ** *p* < 0.01, *** *p* < 0.001). +, −, presence or absence of indicated substance, respectively

NLRP3-mediated IL-1β secretion by human monocyte-derived MΦs requires two distinct signals. The first priming signal involves the upregulation of the NLRP3 inflammasome components and that of the pro-IL-1β. The second signal promotes the assembly of the complex, the activation of caspase-1 enzyme, and subsequently, the processing of IL-1β [35]. To delineate whether the LPS-induced priming signal is affected by ATRA, we determined the protein expression of the inflammasome components. Using the Western blot method, we did not find changes in the expression of the adaptor ASC and the pro-form of caspase-1 enzyme (Figure 2A). Nevertheless, the expression of the NLRP3 sensor and pro-IL-1β substrate was significantly enhanced in the ATRA+LPS-treated samples compared to the LPS-primed ones. Furthermore, we detected a stronger band intensity of cleaved caspase-1 and IL-1β in those samples that were pre-treated with ATRA, indicating that ATRA may also enhance the activity of caspase-1 (Figure 2A).

To find out if ATRA modulates LPS-induced NLRP3 and pro-IL-1β expression at the transcription level, we isolated RNA from the cells at different time points following treatments. Using the quantitative RT-PCR method, we obtained similar results to that of the protein expression, as we detected enhanced mRNA expression of NLRP3 and pro-IL-1β in the ATRA+LPS cells compared to the LPS-treated ones (Figure 2B). These results altogether show that ATRA prolongs LPS-induced IL-1β secretion in part by potentiating LPS-induced NLRP3 and pro-IL-1β expression.

### 3.3. ATRA Alone Enhances NLRP3 but Not Pro-IL-1β Expression

**Figure 3:**
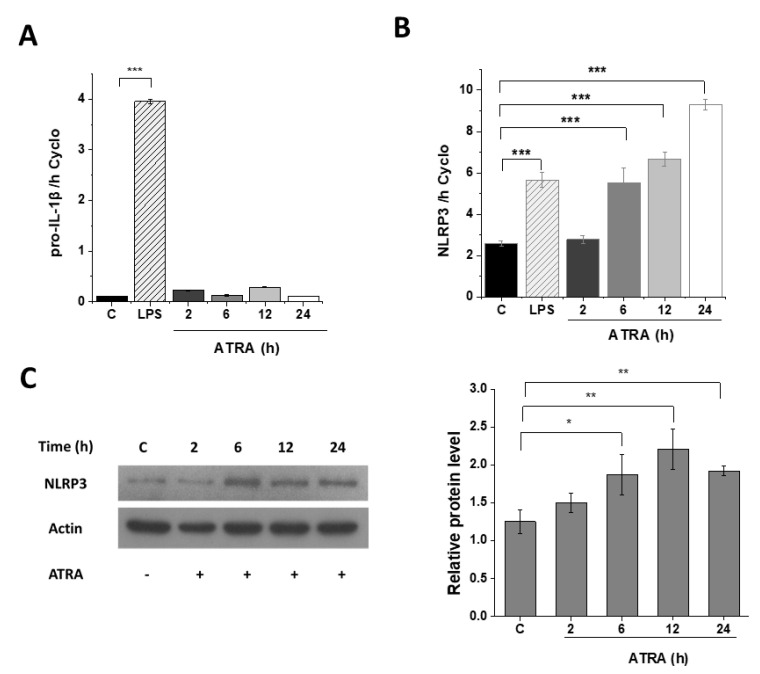
Induction of NLRP3 by ATRA. Relative gene expression of pro-IL-1β and NLRP3 were measured by quantitative RT-PCR. (**A**, **B**) MΦs were treated with ATRA for different time intervals. Here, 6-h LPS-primed cells served as a positive control. (**C**) Representative immunoblot of NLRP3 protein expression of ATRA-treated MΦs as indicated. β-actin was used as the internal control. C, control (6 h mock-treated cells). The data was obtained from at least four healthy donors. All results are shown as means ± SEM. (* *p*< 0.05, ** *p* < 0.01, *** *p* < 0.001). +, −, presence or absence of indicated substance, respectively

Next, we aimed to see whether ATRA alone may serve as a priming signal for NLRP3 inflammasome. Using in silico analysis of a public data base of microarray results [27], we found elevated expression of both NLRP3 and pro-IL-1β in ATRA-stimulated human monocytes, compared to non-stimulated cells (Supplementary Figure S1A). However, using human monocytes, we could not validate these results in an in vitro experiment, as we detected elevated expression of NLRP3, while the expression of pro-IL-1β did not change following ATRA treatment (Supplementary Figure S1B). To elucidate whether ATRA alone has an effect on the expression of NLRP3 and pro-IL-1β in MΦs, we stimulated the cells solely with ATRA, and studied their expressions using Q-RT-PCR and Western blot methods. Similar to our findings in monocytes, we did not observe changes in the expression of pro-IL-1β (Figure 3A); however, the expression of NLRP3 was significantly, and time-dependently upregulated both at the mRNA (Figure 3B) and protein levels (Figure 3C). These results indicate that although ATRA alone is able to enhance the expression of the NLRP3 sensor component of the inflammasome, this stimulus is not enough to trigger the expression of the inflammasome substrate pro-IL-1β.

### 3.4. ATRA Modifies Signal Transduction Pathways Required for Inflammasome Priming

**Figure 4:**
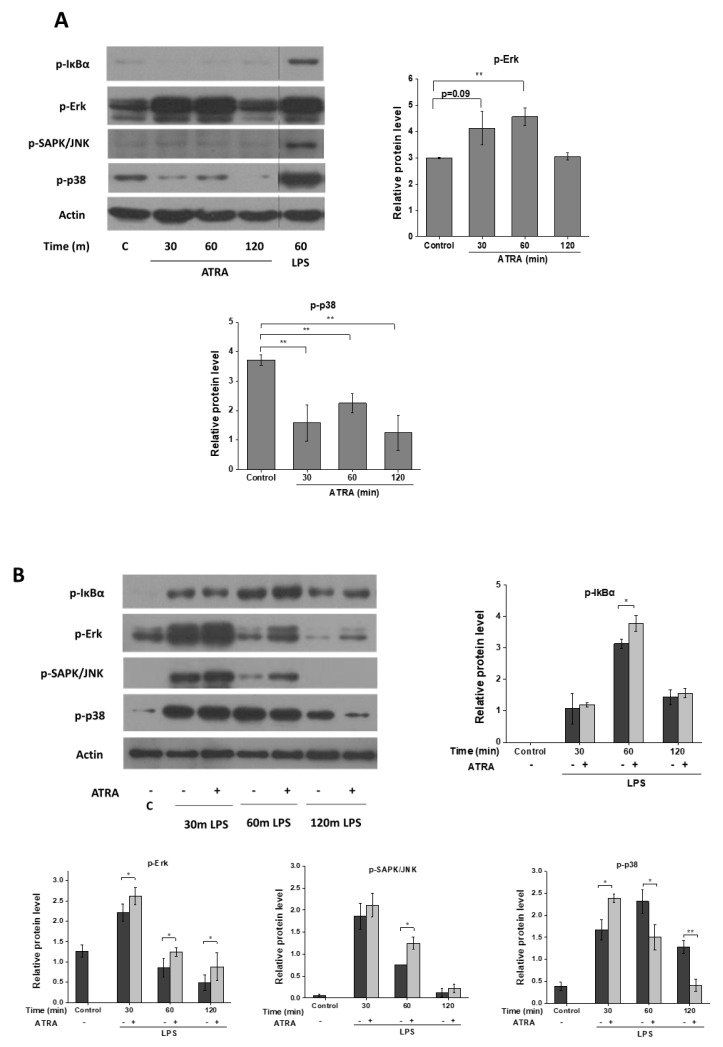
ATRA mediates signal transduction changes in MΦs. (**A**) Representative immunoblots of phosphorylated IkBα, Erk, SAPK/JNK, and p38 from whole-cell lysates after ATRA treatment for the indicated time points. Here, 60-min LPS-primed cells served as a positive control. (**B**) MΦs were pre-incubated with ATRA (1 µM) prior to LPS priming for the indicated time, and phosphorylated IkBα, Erk, SAPK/JNK, and p38. β-actin was used as the internal control. C, control (mock-treated cells). The data were obtained from at least four healthy donors. All results are shown as means ± SEM. (* *p* < 0.05, ** *p* < 0.01). +, −, presence or absence of indicated substance, respectively

ATRA exerts its effect through RAR nuclear receptors [14]. Besides direct regulation of gene expression, RAR can be located in the lipid rafts of cell membranes, and the ligation of the receptor induces the rapid activation of signaling cascades, like p38 and ERK [19,20,21,36]. To study whether ATRA modifies signaling pathways required for NLRP3 inflammasome priming, MΦs were treated with ATRA alone or in combination with LPS, and cell lysates were used for Western blot analysis of signal transduction pathways. Interestingly, while we did not detect changes in IkB-α and SAPK/JNK pathways, phosphorylation of Erk was significantly enhanced and that of the p38 was attenuated following ATRA treatment (Figure 4A). Thereafter, we explored whether ATRA modifies signaling pathways activated by LPS, and we found that it slightly augmented the LPS-induced phosphorylation of IkB-α, and significantly prolonged LPS-induced Erk and SAPK/JNK phosphorylation, while having a moderate but significant inhibitory effect on p38 phosphorylation (Figure 4B). These results show that ATRA modifies cytoplasmic signaling pathways that reportedly play an important role in NLRP3 priming. 

### 3.5. ATRA Inhibits the LPS-Induced AKT/mTOR Signaling Pathway

**Figure 5:**
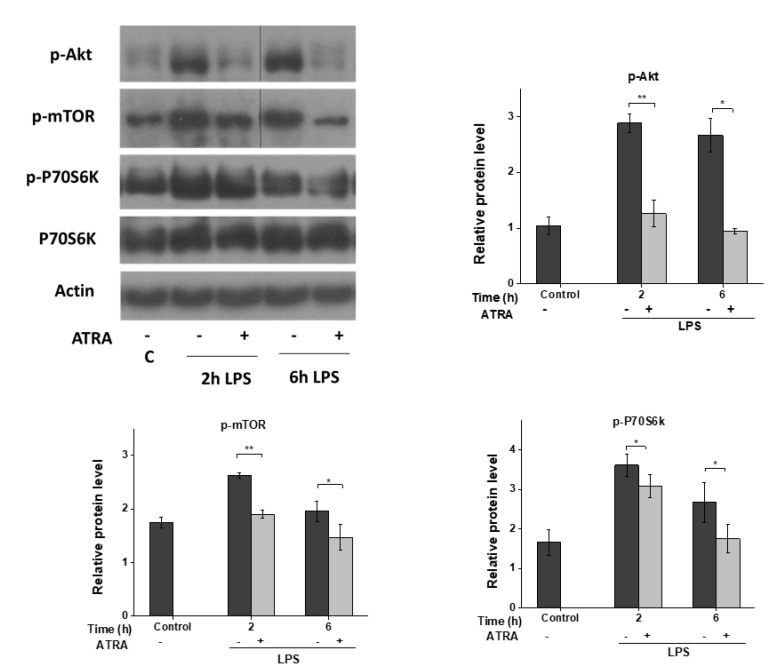
ATRA mediates signal transduction changes in MΦs. Representative immunoblots of phosphorylated Akt, mTOR, P70S6K, and total P70S6K from whole-cell lysates. MΦs were pre-incubated with ATRA (1 µM) prior to LPS priming for the indicated time; then, whole-cell lysates were used for Western blot. β-actin was used as the internal control. C, control (mock-treated cells). The data was obtained from at least four healthy donors. All results are shown as means ± SEM. (* *p* < 0.05, ** *p* < 0.01). +, −, presence or absence of indicated substance, respectively

Toll-like receptor (TLR)-induced signaling and cytokine secretion is also affected by the Akt/mTOR signaling pathways [37]. Furthermore, mTOR was shown to regulate inflammasome function by inhibiting caspase-1 processing [38]. For this reason, we sought to determine whether ATRA has any modulatory effect on the LPS-activated mTOR pathway. We found that LPS stimulation of MΦs induced Akt phosphorylation and activated mTOR as well as its downstream target p70S6K (Figure 5). Surprisingly, however, ATRA pre-treatment almost completely abolished LPS-induced phosphorylation of Akt; furthermore, the subsequent downstream signaling pathways, including mTOR and p70S6K, were also attenuated.

### 3.6. ATRA Attenuates Secretion of LPS-Induced IL-10

**Figure 6:**
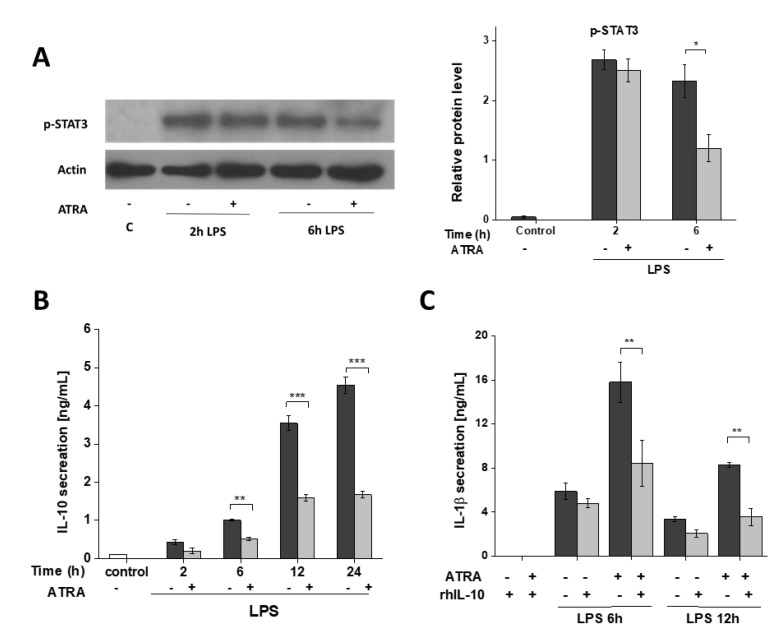
ATRA modulates LPS-induced IL-10 secretion and STAT3 activation in MΦs. MΦs were pre-incubated with ATRA (1 µM) prior to stimulation with LPS (100 ng/mL) for the indicated time. (**A**) Representative immunoblots of phosphorylated STAT3 from whole-cell lysates. β-actin was used as the internal control. (**B**) The secretion of IL-10 was assessed by ELISA from cell culture supernatants. (**C**) The cells were pre-treated with recombinant human IL-10 (rhIL-10) (100 ng/mL) 1 h before LPS priming and subsequently incubated with ATP (5 mM) for 45 min. Then, the cell culture supernatants were collected, and the secretion of IL-1β was assessed by ELISA. C, control (mock-treated cells). The data were obtained from at least four healthy donors. All results are shown as means ± SEM. (* *p*< 0.05, ** *p* < 0.001, *** *p* < 0.001). +, −, presence or absence of indicated substance, respectively

STAT3 is a potential downstream target of mTORC1 signaling in MΦs [39]; additionally, it is one of the most efficient regulators of IL-10, a master anti-inflammatory cytokine [37,40]. For this reason, next we aimed to study whether inhibition of the Akt/mTOR pathways by ATRA has any effect on STAT3 signaling, and eventually on IL-10 secretion in the LPS-activated cells. Challenge with LPS highly induced the phosphorylation of STAT3, while ATRA significantly downregulated this activation (Figure 6A). Importantly, we also observed a significant attenuation in the LPS-induced IL-10 secretion at each time-point in the presence of ATRA (Figure 6B). As IL-10 mediates inhibition of proinflammatory cytokine secretion [41], we aimed to see whether IL-10 can reverse the augmenting effect of ATRA on IL-1β secretion. Application of recombinant human IL-10 to the ATRA+LPS-treated cells significantly decreased IL-1β secretion. These results suggest that the enhanced IL-1β secretion of LPS-activated cells following ATRA treatment is mediated, in part, by the attenuated STAT3/IL-10 signaling axis. These results also suggest that in LPS-activated MΦs, ATRA predominates the proinflammatory characteristics over of the anti-inflammatory ones.

### 3.7. ATRA Mediates a Metabolic Shift Towards Glycolysis in LPS-Stimulated MΦs

**Figure 7:**
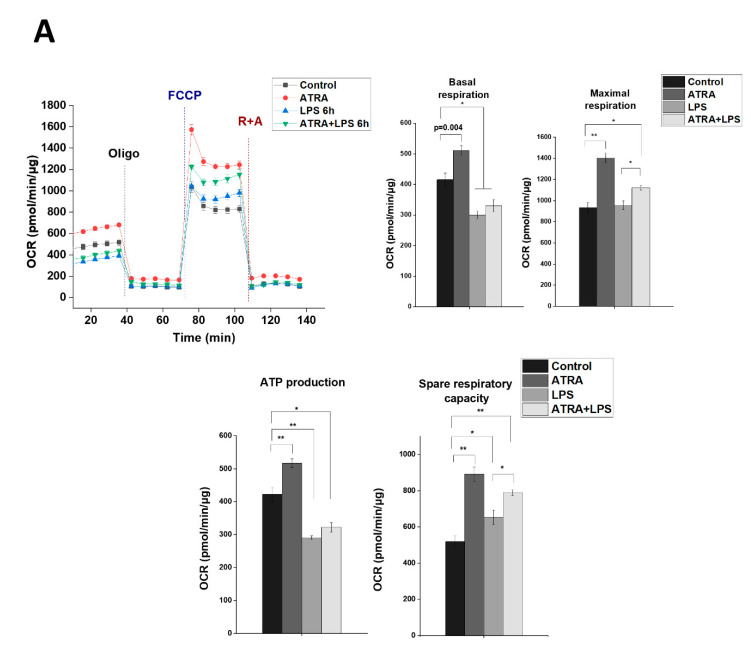

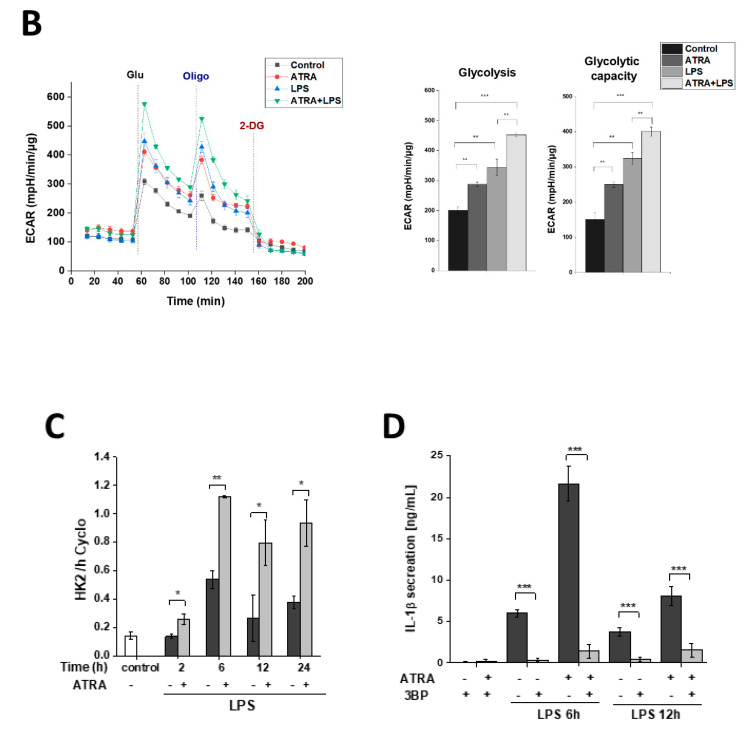
ATRA mediates metabolic changes in LPS-primed MΦs. The cells were pre-treated with or without ATRA, then primed with LPS for 6 h, and subsequently subjected to the mitochondria stress test using a Seahorse XF96 Analyzer. (**A**) Real-time kinetics measurement of the oxygen consumption rate (OCR) during sequential treatment with oligomycin (Oligo), carbonylcyanide-4-(trifluoromethoxy) phenylhydrazone (FCCP), and antimycin A + rotenone (A+R). Representative results are shown. Bar graphs represent the calculated basal and maximal OCR, and ATP-coupled respiration and spare respiratory capacity. (**B**) Real-time kinetics measurement of the extracellular acidification rate (ECAR) after sequential treatment of glucose (Glu), oligomycin (Oligo), and 2-deoxyglucose (2-DG). Representative results are shown. Bar graphs represent calculated ECAR and glycolytic capacity obtained from the glycolytic stress test. Wave Desktop software was used for data analysis. (**C**) Relative gene expression of HK2 was measured by qPCR. The expression was normalized to the reference gene (human cyclophilin) expression. (**D**) MΦs were pretreated with 3-bromopyruvate (3BP) (80 µM) 1 h before LPS priming and subsequently incubated with ATP (5 mM) for 45 min., and then the cell culture supernatants were collected, and the secretion of IL-1β was assessed by ELISA. C, control (mock-treated cells). The data was obtained from at least four healthy donors. All results are shown as means ± SEM. (* *p* < 0.05, ** *p* < 0.01, *** *p* < 0.001). +, −, presence or absence of indicated substance, respectively

mTOR has a central regulatory role in several vital cellular functions, such as cell growth and energy metabolism [42]. In order to elucidate if ATRA could affect mitochondrial functions under LPS challenge, MΦs were subjected to LPS stimulation (for 6 h) in the absence or presence of ATRA. Cells were then analyzed for changes in the mitochondrial rate of oxygen consumption (OCR) and the rate of extracellular acidification (ECAR), as a measure of oxidative phosphorylation (OXPHOS) and glycolysis, respectively. Interestingly, we found that ATRA treatment alone significantly enhanced the studied mitochondrial functions; however, ATRA pre-treatment was not able to recover the LPS-induced downregulation of basal respiration and ATP production (Figure 7A). Importantly, nevertheless, ATRA pre-treatment significantly enhanced the LPS-attenuated OCRs of maximal respiration and spare respiratory capacity (SRC), parameters that indicate the fitness of mitochondria, suggesting that ATRA has a protective role in the mitochondria (Figure 7A). 

Regarding glycolysis, interestingly, we found that both ATRA and LPS individually significantly enhanced ECARs in the MΦs. Nevertheless, importantly, we detected a significantly higher glycolysis and glycolytic capacity in the ATRA+LPS-treated cells compared to the LPS- or ATRA-treated ones, showing an additive effect (Figure 7B). Consistent with these results, ATRA pre-treatment of LPS-activated MΦs induced a significant upregulation of the expression of hexokinase 2 (HK2), the rate-limiting enzyme and an indicator of the glycolytic pathway [43] (Figure 7C). To determine whether HK2 activity indeed affects IL-1β secretion, we treated MΦs with 3-bromopyruvate (3BP), a specific inhibitor of HK2. Using IL-1β ELISA, we found that 3BP significantly attenuated the secreted level of IL-1β cytokine (Figure 7D). Altogether, these results indicate that in LPS-activated human MΦs, ATRA triggers a rapid metabolic shift towards glycolysis, which, in part, drives the secretion of an elevated amount of IL-1β.

## 4. Discussion

Retinoic acid is a metabolite of vitamin A, and a major regulator of homeostasis and immune responses of epithelial tissues and the mucosa [44]. Vitamin A is obtained from the diet and following metabolism it is stored in the liver as retinol [45]. When it is released into the bloodstream, it is absorbed by target tissues and cells, and thereafter metabolized to different forms of retinoic acid (RA), of which, physiologically, all-trans RA (ATRA) is the most abundant [46]. The effects of ATRA on myeloid cells are mainly studied in intestinal mucosal DCs and MΦs. It was shown that at steady-state conditions, ATRA is produced by epithelial cells and stromal cells, and instructs mucosal DCs and resident MΦs to develop inflammatory tolerance [47]. During infection, mucosal DCs produce proinflammatory cytokines and drive the differentiation of T effector cells, while mucosal MΦs develop inflammatory anergy in spite of their phagocytic activity [47], thereby maintaining proper mucosal homeostasis [17].

Additionally, ATRA is also produced by murine bone marrow-derived MΦs or human monocyte-derived MΦs following activation [48]; also, it can be absorbed from the circulation, thus ATRA regulates local inflammatory responses in non-mucosal tissues where infiltrating monocytes differentiate in situ into MΦs during inflammation. Importantly, it was shown that in contrast to mucosal MΦs, ATRA suppressed nitric oxide synthesis, IL-12, and TNFα cytokine secretion, while enhancing IL-10 production in LPS-activated murine peritoneal MΦs and cord blood mononuclear cells [49,50,51]. Furthermore, it was also reported that ATRA enhanced LPS-induced IL-1β expression in human alveolar MΦs and THP-1 cells [31,32].

**Figure 8:**
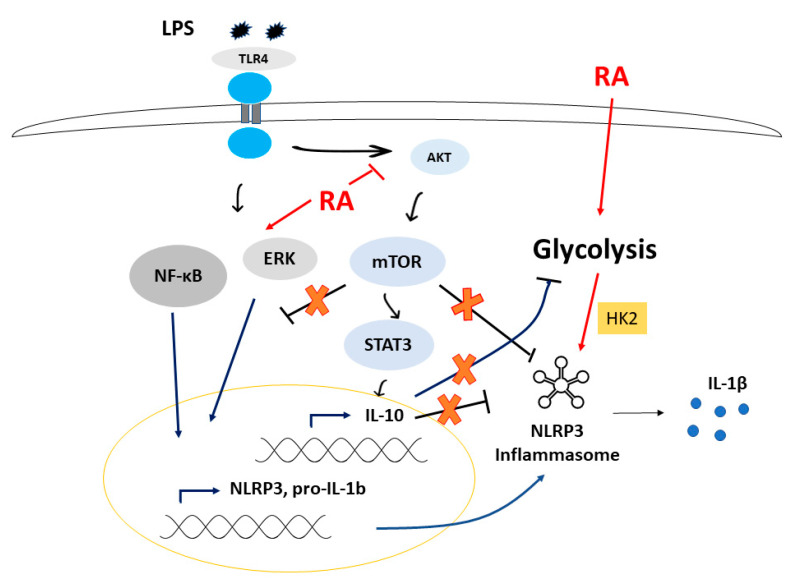
Proposed model for the effects of ATRA.

Using LPS-activated human monocyte-derived MΦs, we found that while the secretion of TNFα was not affected, IL-6 and IL-1β secretion was significantly augmented by ATRA. IL-1β is a conductor proinflammatory cytokine with a versatile function and has coordinating roles in both innate and adaptive immune responses [52]. As a safeguard mechanism, in human monocyte-derived MΦs, the production of IL-1β following LPS activation is highly regulated and requires both priming and activating stimuli of NLRP3 inflammasome [35]. Our results show for the first time that ATRA prolongs LPS-induced IL-1β secretion by enhancing both the priming and activation of NLRP3 inflammasome (Figure 8).

We found that ATRA treatment significantly enhanced the LPS-induced NLRP3 and pro-IL-1β expression; moreover, we showed that ATRA alone is also capable of inducing the expression of the NLRP3 sensor. ATRA exerts its effect through RAR nuclear receptors that regulate the expression of their target gene by binding to the RAR response element (RARE) [53]. Our results may suggest that ATRA, in part, induces a direct RAR-driven transcription of NLRP3. Although, in an in silico analysis, we indeed found putative consensus sequences for RAR binding in the NLRP3 gene (data not shown). This should be interpreted with caution, and the verification would require further elaborate and complex genomic studies. In the past decade, several studies have proved that the sole presence of a transcription factor binding site is not satisfactory evidence for its activity [54], as other criteria, such as neighboring DNA sequences, trans-acting elements, transcription co-factors’ availability, cell lineage-determining factors, or the cross-talk of transcription factors during a given cell activation, should all be considered [55,56]. Nevertheless, though the expression of NLRP3 is usually mediated through specific signal transduction pathways triggered by cell membrane-located receptors like TLRs or cytokine receptors [57], RAR would not be the first nuclear receptor announced as a regulator of NLRP3 transcription. Response elements for VDR, PPARγ, and RORγ have also been found at the promoter region of NLRP3 and pro-IL-1β genes, and ligation of these receptors resulted in either an enhanced or attenuated expression [58,59,60].

However, the function of ATRA is not restricted to genomic effects. Importantly, an extranuclear function of RAR has also been reported, as it was shown that ligated RAR modulates the activation of signaling pathways in the cytoplasm by interacting with Akt, p38, and ERK [19,20,21]. Here, we showed that ATRA alone significantly downregulated p38 signaling, while it upregulated ERK signaling. Moreover, ATRA also attenuated the LPS-induced p38 pathway, while augmenting the NF-kB, ERK, and JNK pathways. Our group along with other laboratories have previously reported that these pathways are important regulators of NLRP3 and pro-IL-1b expression [34,57]. Even though further studies are required to describe the details of the regulatory mechanisms, we propose that ATRA regulates priming of NLRP3 inflammasome via multiple pathways.

Besides priming, the assembly and activation of NLRP3 inflammasome is also triggered by a wide range of intracellular or extracellular stimuli [61]. Our results show that the LPS-activated Akt/mTOR signaling is significantly downregulated by ATRA. mTOR inhibitors have shown promising results in advanced clinical trials against certain malignancies [62], as Akt/mTOR may limit proinflammatory, and induce anti-inflammatory responses [63]. Importantly, mTOR signaling is a pivotal negative regulator of NF-kB signaling and caspase-1 activation but is also a positive regulator of IL-10 secretion via the STAT3 pathway, providing a feedback loop to limit excessive inflammation, including IL-1β secretion in myeloid cells [37,38,64]. Our results show that STAT3 phosphorylation is significantly attenuated following ATRA treatment; furthermore, we observed a dramatic inhibition in the secretion of IL-10 anti-inflammatory cytokine of the primed MΦs. Using recombinant IL-10, the ATRA-enhanced IL-1β secretion was indeed significantly decreased in the LPS-activated cells. Based on our results, we suggest that ATRA treatment of primed MΦs leads to reduced secretion of IL-10 via attenuated Akt/mTOR/STAT3 signaling, thus alleviating the inhibitory feedback loop on IL-1β, resulting in prolonged IL-1β secretion. 

mTOR is also a sensor and a key regulator of energy metabolism, and changes in the metabolic pathways are a hallmark of activation and polarization of MΦs [65]. In general, enhanced glycolysis is observed during inflammatory responses, while mitochondrial oxidative phosphorylation is more characteristic of anti-inflammatory responses [66]. While it is clear that metabolic changes, including glycolysis, significantly modulate and regulate inflammasome activation, reported mechanisms are contradictory, and do not provide detailed evidence. Some studies suggest that undisturbed glycolysis is required for the activation of NLRP3 inflammasome [67,68,69,70], whereas others demonstrated that inhibition of glycolytic enzymes results in robust NLRP3 inflammasome activation [71,72]. In this current study, we showed that ATRA shifts the metabolic pathways towards glycolysis and increases HK2 expression in LPS-primed MΦs. Furthermore, inhibition of HK2 resulted in the attenuation of IL-1β secretion. Many glycolytic enzymes have already been shown to control the NLRP3 activation status [67,71,73]. Hexokinase (HK), the first enzyme in glycolysis, is assumed to be an important regulator of NLRP3 inflammasome activation [67]. HK2 is a constitutively active enzyme that is recruited to, and bound to the mitochondrial outer membrane to drive glycolysis [74]. In the case of excess glucose, HK2 activity leads to glycolytic overload, resulting in the accumulation of glucose-6-phosphate (G6P) [75]. G6P is an activator of the sugar sensor Mondo A/Mlx transcription factors that preferentially drive TXNIP expression [76], an activator of NLRP3 inflammasome [77]. Importantly, ATRA was reported to enhance glucose transporter (GLUT) expression and glucose uptake [78,79], while IL-10 was shown to limit glucose uptake and glycolytic flux to sustain OXPHOS [80]. Based on our results and the available reports in the field, we hypothesize that ATRA enhances glucose uptake to drive enhanced glycolysis, eventually resulting in augmented NLRP3 activation in LPS-primed MΦs. 

## 5. Conclusions

Our data demonstrate a novel mechanistic role for ATRA in the NLRP3 inflammasome-mediated innate immune response. We showed that ATRA enhances and prolongs IL-1β secretion of LPS-activated human monocyte-derived MΦs by augmenting both the priming and the activating signals (Figure 8). Epidemiological studies have proved the association between vitamin A and adequate immune response in bacterial infections [81]. Vitamin A deficiency may result in increased susceptibility to various bacterial and viral infections, such as tuberculosis and malaria [82,83,84,85]. Importantly, it was also reported that supplementation with vitamin A or retinoids reduced infectious complications, and improved immune responses, in part, by the activation of myeloid cells and changing their cytokine production [29,86]. Additionally, it was also speculated that enhanced IL-1 β levels could contribute to the anticancer effects of vitamin A by potentiating macrophage-dependent tumor defense mechanisms [87].

Based on our results, we suggest that in infectious conditions, ATRA boosts IL-1β, a conductor proinflammatory cytokine, production via the NLRP3 inflammasome-mediated pathway in monocyte-derived macrophages. This observation may partly explain the improved inflammatory responses observed following retinoid supplementation. Although the in vivo relevancy of our findings requires further investigation, our results may provide potential therapeutic tools for conditions where inflammatory responses should be further potentiated, such as in infectious diseases and in antitumor therapies.

## Supplementary Materials

The following are available online at https://www.mdpi.com/2073-4409/9/7/1591/s1, Figure S1: Induction of NLRP3 expression by ATRA on monocytes. (A) In silico analysis results for human monocytes treated with ATRA obtained from gene expression omnibus (GEO) database, accession number: GSE46268. (B) Relative gene expression of IL-1β and NLRP3 were measured by quantitative-RT-PCR. Isolated human monocytes were plated for 2 h then treated with ATRA or left untreated for 24 h. Data were obtained from at least three healthy donors. All results are shown as means ± SD. (* *p* < 0.05, ** *p* < 0.01).
