# Supplementary material for: All-Trans Retinoic Acid Enhances both the Signaling for Priming and the Glycolysis for Activation of NLRP3 Inflammasome in Human Macrophage
Source: Cells. 2020 Jul 1;9(7):1591. doi: 10.3390/cells9071591 (PMC7407903; doi:10.3390/cells9071591)
Supplement: Supplementary file 1 [file cells-09-01591-s001.pdf]

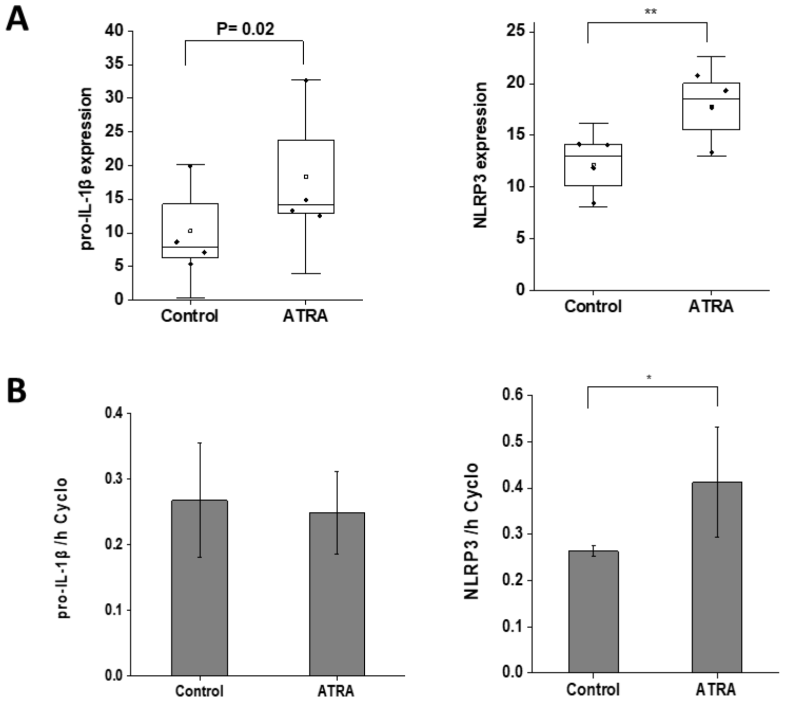

Figure S1. Induction of NLRP3 expression by ATRA on monocytes. **(A)** In silico analysis results for human monocytes treated with ATRA obtained from gene expression omnibus (GEO) database, accession number: GSE46268. **(B)** Relative gene expression of IL-1β and NLRP3 were measured by quantitative-RT-PCR. Isolated human monocytes were plated for 2 h then treated with ATRA or left untreated for 24 h. Data were obtained from at least three healthy donors. All results are shown as means ± SD. (*p< 0.05, **p < 0.01).
